# Vestibular disorder and autonomic dysfunction

**DOI:** 10.1016/S1808-8694(15)31395-1

**Published:** 2015-10-17

**Authors:** Letícia Boari, Adriana Gonzaga Chaves, Fernando Freitas Ganança, Mário Sérgio Lei Munhoz

**Affiliations:** 1 Master in otorhinolaryngology, graduate course of the Santa Casa de Sao Paulo. Fellow in otoneurology, Department of Otorhinolaryngology and Head & Neck Surgery, UNIFESP. ENT specialist; 2 Master’s degree student in otorhinolaryngology, graduate course of the UNIFESP ENT specialist; 3 Master and doctor in otorhinolaryngology, UNIFESP-EPM. Professor of otoneurology, Department of Otorhinolaryngology and Head & Neck Surgery, UNIFESP-EPM; 4 Livre Docente (habilitation) professor, UNIFESP-EPM. Head of the otoneurology discipline, Department of Otorhinolaryngology, UNIFESP-EPM. UNIFESP-EPM

**Keywords:** autonomic dysfunction, dizziness, vertigo, vestibular disease

## INTRODUCTION

Dizziness is a common symptom in medical practice, and may be associated with over 300 diseases. Although most cases of dizziness are related to vestibular system dysfunction, some result from involvement of other parts of the body.^1^ Recent studies have shows that there is an association between auditory/vestibular symptoms and autonomous nervous system dysfunction, suggesting that this may be a cause of certain vestibular diseases.^2^, ^3^, ^4^, ^5^

## CASE REPORT

CLBP, a female patient aged 47 years presented with non-rotating dizziness, a loss of balance lasting from 3 to 5 minutes for the past three years. Dizziness disappeared spontaneously upon lying down. Other accompanying symptoms included nausea, vomiting, darkened vision and cold sweating. Dizziness occurred two to three times monthly, usually due to abrupt bodily movements, such as standing up. The patient reported that she had already fainted after a crisis of dizziness.

There was no hearing loss or aural fullness. The patient reported continuous non-pulsatile tinnitus during the past three years that worsened during dizziness crises. There were no other issues in her medical history.

The patient reported a balanced diet, but with long intervals between meals.

The otoneurological exam revealed no findings. Laboratory exams were within normal limits.

**Figure 1:**
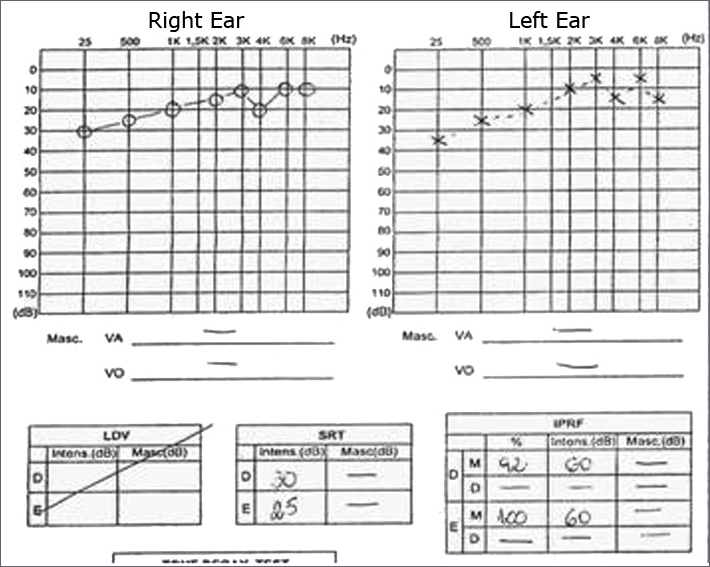
Pure tone and voice audiometries of the patient prior to therapy.

Pure tone and voice audiometry (Figure 1) showed mild sensorineural hearing loss at low frequencies (250 - 500 Hz); immitance testing of the stapedial reflex showed bilateral recruitment. Vectoelectronystagmography results suggested bilateral irritative peripheral vestibular syndrome.

The patient was oriented about her diet and referred to the cardiology department. Various tests found only an altered tilt test. The hypothesis was neurocardiogenic syncope, and therapy was started by the arrhythmia team.

The patient has been taking propranolol, fludrocortisone and fluoxetine for four months, and reports significant improvement from dizziness, syncope and tinnitus.

An audiometry was within normal limits.

## DISCUSSION

Recent studies correlating vestibular conditions with autonomous nervous system disorders have been published. The current prevalence of this condition is low, although there is the likelihood that it is underdiagnosed.

Staab et al. reviewed 1,400 cases of patients with dizziness and reported that the prevalence of effort-induced dizziness was 0.64%, provoked by malfunction of the autonomous nervous system.^4^

Pappas assessed 113 cases of patients diagnosed with vestibular disorders and dysautonomia, and reported that 58 cases had been previously treated as having Ménière’s disease. Of these patients, 9% had improved, symptoms were unchanged in 34%, and auditory/vestibular symptoms worsened in 57%. After therapy for dysautonomia was started, 86% of cases improved and 14% were unchanged; no cases worsened.^5^

These findings underline the importance of physicians being attentive to clinical pictures that do not progress satisfactorily; the investigation may be insufficient, with consequences in the treatment.

The case report illustrates the probable correlation between vestibular disorders and dysautonomia. No other likely cause for the patient’s symptoms, except for the neurocardiogenic syncope, was found. Dietary deficiencies may increase vestibular dysfunction, but clinical improvement after therapy for dysautonomia suggests that this condition had provoked the otological and vestibular findings, as has been reported by other authors.^4^, ^5^

## FINAL COMMENTS

Further studies are needed to clarify the connection between dysautonomia and vestibular disorders, especially its physiology and pathology. There is enough evidence to suggest that dysautonomia is an important cause of dizziness, and possibly other otoneurological conditions.
